# The value of a metabolic reprogramming-related gene signature for pancreatic adenocarcinoma prognosis prediction

**DOI:** 10.18632/aging.104134

**Published:** 2020-11-20

**Authors:** Zhen Tan, Yubin Lei, Jin Xu, Si Shi, Jie Hua, Bo Zhang, Qingcai Meng, Jiang Liu, Yiyin Zhang, Miaoyan Wei, Xianjun Yu, Chen Liang

**Affiliations:** 1 Department of Pancreatic Surgery, Fudan University Shanghai Cancer Center, Shanghai 200032, China; 2 Department of Oncology, Shanghai Medical College, Fudan University, Shanghai 200032, China; 3 Shanghai Pancreatic Cancer Institute, Shanghai 200032, China; 4 Pancreatic Cancer Institute, Fudan University, Shanghai 200032, China

**Keywords:** pancreatic adenocarcinoma, metabolic reprogramming

## Abstract

Pancreatic ductal adenocarcinoma (PDAC) is one of the most fatal malignancies worldwide. Extensive enhancement of glycolysis and reprogramming of lipid metabolism are both associated with the development and progression of PDAC. Previous studies have suggested that various gene signatures could convey prognostic information about PDAC. However, the use of these signatures has some limitations, perhaps because of a lack of knowledge regarding the genetic and energy supply backgrounds of PDAC. Therefore, we conducted multi-mRNA analysis based on metabolic reprogramming to identify novel signatures for accurate prognosis prediction in PDAC patients. In this study, a three-gene signature comprising MET, ENO3 and CD36 was established to predict the overall survival of PDAC patients. The three-gene signature could divide patients into high- and low-risk groups by disparities in overall survival verified by log-rank test in two independent validation cohorts and could differentiate tumors from normal tissues with excellent accuracy in four Gene Expression Omnibus (GEO) cohorts. We also found a positive correlation between the risk score of the gene signature and inherited germline mutations in PDAC predisposition genes. A glycolysis and lipid metabolism-based gene nomogram and corresponding calibration curves showed significant performance for survival prediction in the TCGA-PDAC dataset. The high-risk designation was closely connected with oncological signatures and multiple aggressiveness-related pathways, as determined by gene set enrichment analysis (GSEA). In summary, our study developed a three-gene signature and established a prognostic nomogram that objectively predicted overall survival in PDAC. The findings could provide a reference for the prediction of overall survival and could aid in individualized management for PDAC patients.

## INTRODUCTION

Pancreatic ductal adenocarcinoma (PDAC) is one of the most invasive solid malignancies and could become the second leading source of cancer-related deaths in the United States. Surgical resection, which is the only established therapy, significantly enhances the five-year survival rate to 20–30%. However, fewer than 20% of all PDAC patients are eligible for resection since most patients are diagnosed with advanced-stage disease featuring metastasis. However, patients with PDAC in the same TNM stage can differ in overall survival (OS), perhaps because of the complex desmoplastic microenvironment and the different molecular characteristics of PDAC. Thus, new strategies and better signatures are needed to help predict prognosis and subsequently improve individualized treatment for PDAC patients.

Recent advances in gene chips and high-throughput next-generation sequencing technology have modified the transcriptomic research landscape and demonstrated that mRNA prognostic signatures can help to predict OS in PDAC [1]. Numerous bioinformatics analyses have been conducted to elucidate molecular mechanisms and direct clinical practice [2]. Raman et al. developed a five-gene prognostic model that accurately predicted OS from a PDAC dataset in The Cancer Genome Atlas (TCGA) (the TCGA-PDAC dataset) using Cox proportional hazards regression analysis [3]. Similarly, Yan et al. revealed a four-gene signature (with LYRM1, KNTC1, IGF2BP2, and CDC6) that is significantly related to the progression of pancreatic cancer through the same method [4]. However, this method is not suitable for high-dimensional microarray data because of the limitation of overfitting. The least absolute shrinkage and selection operator (LASSO) regression method does not have this class of limitation and has been broadly used for optimized selection of genes [5]. Notably, PDAC is characterized by an incredibly nutrient-deficient and hypoxic environment caused by vascular disturbances, desmoplastic reactions and unrestrained growth. Oncogenic activation of Kirsten rat sarcoma 2 viral oncogene homolog (KRAS) and mutations in other tumor suppressor genes promote abnormal mitochondrial metabolism and enhance glycolysis and lipid metabolism since these genes are intrinsic components of metabolic plasticity [6]. Reprogramming of metabolic pathways, including enhancement of lipid metabolism and glycolysis, has emerged as a hallmark of cancer [7, 8]. The most common transformation is enhancement of glycolysis, which enables vigorous growth of cells by generating a large variety of substrates and facilitates invasion and migration by affecting glycolytic enzymes to improve the supply of ATP [9, 10]. Biosynthesis, glycosylation and redox homeostasis are also linked to intermediates related to glycolysis. Overexpression of glycolytic enzymes and increased lactate production in PDAC cells promote metastatic colonization of distant organs and tumor angiogenesis by regulating the invasion-metastasis cascade [11]. Fatty acids (FAs) and glycerol are key molecules released by adipocytes and are the main precursors of the lipids used as energy sources by cancer cells. As a result, cancer-associated adipocytes play a role in providing energy for cancer cells since they supply sufficient FAs and lipids to affect the metabolism of tumor cells and promote the growth of malignant tumors. FAs are also used by cells to synthesize membranes and to generate signaling molecules that stimulate the proliferation and invasion of cancer cells. Previous studies have sought to delineate correlations between lipid remodeling and the progression of invasive malignant biological behavior in PDAC. Lipid breakdown and fibrotic changes in the tumor microenvironment enhance the levels of FAs.

In this study, we performed a systematic and comprehensive gene signature discovery and validation effort, with consideration of various components of metabolic reprogramming, to develop a multi-gene model for the robust prediction of the prognoses of PDAC patients. A prognostic nomogram consisting of the identified gene signature and clinical prognostic factors was also created for prediction of OS. The molecular mechanism and inherited germline mutations of the gene signature were also investigated. All of these findings could provide a reference for improved prediction of OS and could aid in the individualized management of PDAC patients.

## RESULTS

### Identification of DEGs from public datasets

**Figure 1:**
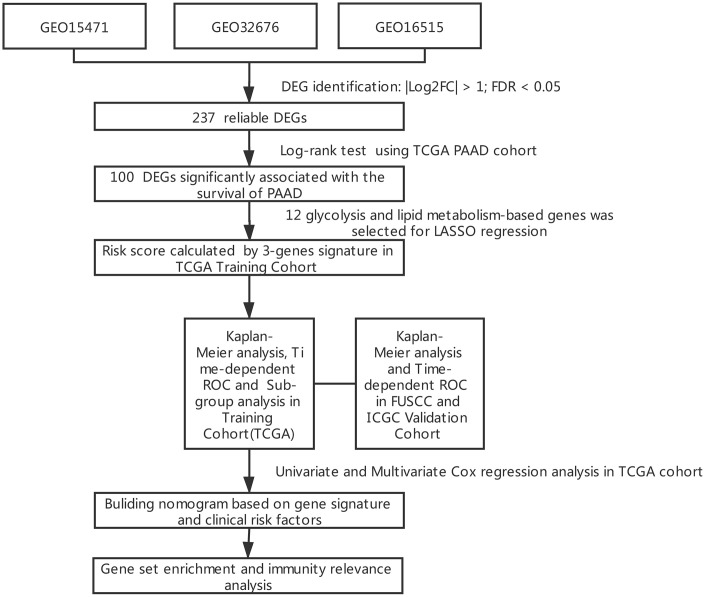
**Flowchart presenting the process of establishing the gene signature in this study.**

**Figure 2:**
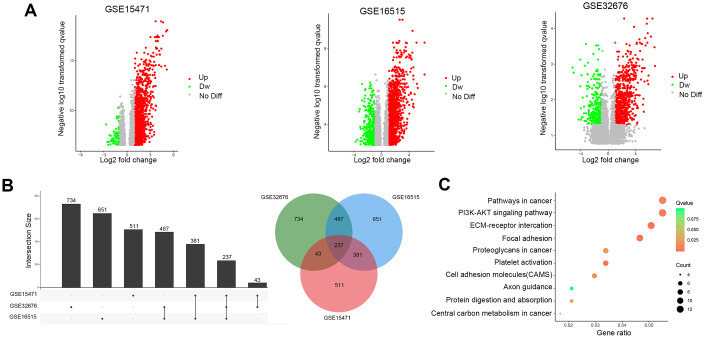
**Identification of DEGs in pancreatic cancer between tumor and paracancerous tissues.** (**A**) Volcano plots of DEGs in the 3 indicated datasets. (X-axis: log2(FC); Y-axis: -log10(FDR) for each gene. Genes with FDR <0.01 and FC >1 or <-1 were considered as DEGs in each series. Blue: down-regulated genes; Gray: non-differential genes; Red: up-regulated genes). (**B**) Upset Venn diagrams of the DEGs identified in 3 GEO datasets. (**C**) Top 10 enriched KEGG pathways of the DEGs.

The results of this study are summarized in a flow chart (Figure 1). The detailed information in three eligible PDAC datasets in the GEO database (GSE15471, GSE16515, and GSE32676) met our criteria. Analysis of these PDAC datasets revealed that 237 differentially expressed genes (DEGs) were shared among the 3 series of comparisons between tumor and adjacent para-cancerous tissues in the Venn and UpSet diagrams; these DEGs were regarded as credible DEGs (Figure 2A, 2B). In addition, KEGG pathway enrichment analyses were performed for these overlapping up- or down-regulated genes. As shown in Figure 2C, the DEGs were most enriched in the ECM-receptor interaction pathway, the PI3K-Akt signaling pathway and numerous energy metabolism-related pathways, such as the proteoglycans in cancer pathway and the central carbon metabolism in cancer pathway.

### Prognostic signature construction

**Figure 3:**
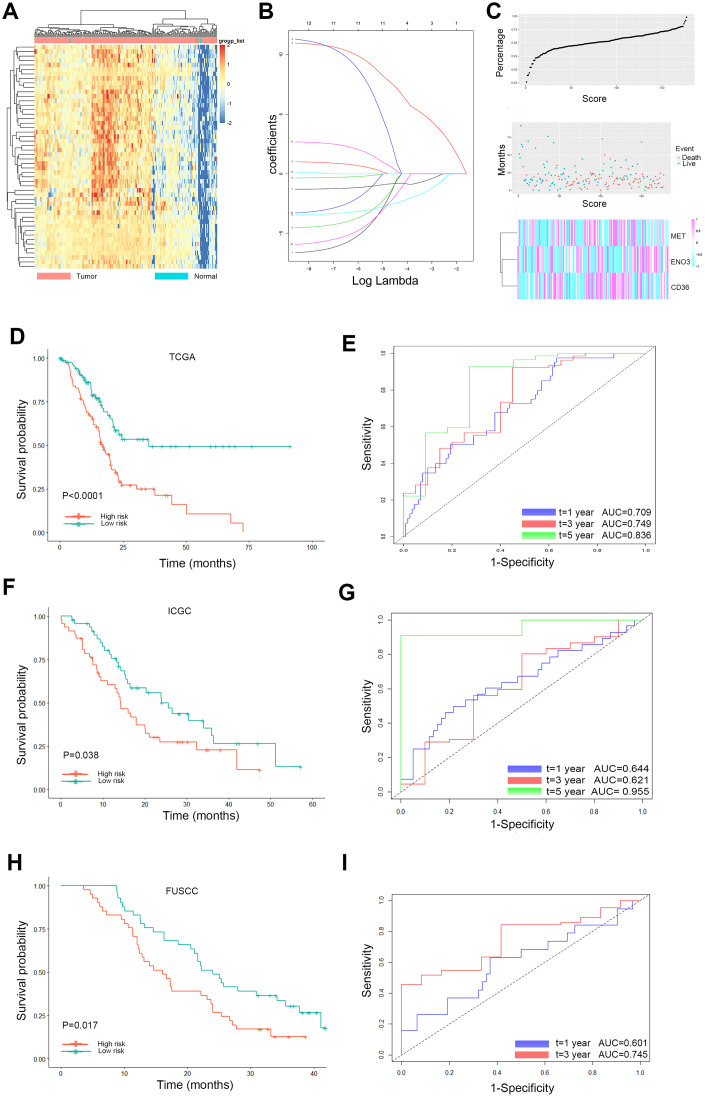
**Lasso analysis and Kaplan-Meier curve for the patients in the TCGA, ICGC and FUSCC cohorts.** (**A**) Representative heatmap of the DEGs significantly related to OS time identified by the log-rank test in the TCGA cohort. (**B**) LASSO coefficient profiles of the 12 glycolysis and lipid metabolism-based genes. LASSO, least absolute shrinkage and selection operator method. (**C**) Risk score analysis of the differentially expressed DEG signatures of PDAC. Risk scores of DEG signatures (top); survival status and duration of cases (middle); low-score and high-score groups for the three genes (bottom). (**D**) The Kaplan-Meier plot (low risk vs. high risk PDAC cases) of 5-year overall survival in the TCGA cohort. (**E**) Time-dependent ROC analyses at 1, 3, and 5 years in the TCGA cohort. (**F**) The Kaplan-Meier plot (low risk vs. high risk PDAC cases) of 5-year overall survival in the ICGC cohort. (**G**) Time dependent ROC analyses at 1, 3, and 5 years in the ICGC cohort. (**H**) The Kaplan-Meier plot (low risk vs. high risk PDAC cases) of 5-year overall survival in the FUSCC cohort (**I**). Time dependent ROC analyses at 1, 3, and 5 years in the FUSCC cohort.

Based on these 237 DEGs and clinical features from the TCGA database, 100 genes significantly related to OS time (P < 0.05) were identified by the log-rank test, as shown in Figure 3A. Twelve glycolysis and lipid metabolism-related genes significantly associated with survival of PDAC were screened (Table 1). To avoid overfitting during the subsequent model construction, LASSO regression was performed to build a prognostic signature, which included twelve genes from the 237 previously identified DEGs. The LASSO coefficient profiles of the twelve genes are presented in Figure 3B. A formula to calculate the risk score for PDAC was derived based on individual three-gene expression levels weighted by regression coefficients, as follows:

**Table 1:** The survival information of the 12 glycolysis and lipid metabolism-based genes.

**gene**	**Overall Survival**		**Disease Free Survival**	
	**HR**	**Log-rank p**	**HR**	**Log-rank p**
PKM	1.7	0.0097	1.6	0.046
MET	2.2	0.00023	2.2	0.00044
HK2	1.5	0.053	1.5	0.084
LDHA	2.2	0.0002	2.1	0.00064
ENO3	0.65	0.045	0.64	0.042
LDLR	1.7	0.012	1.4	0.15
CD36	0.56	0.0053	0.64	0.046
FAS	1.7	0.013	1.2	0.48
SDC1	1.6	0.018	1.8	0.011
SDC4	1.7	0.015	1.8	0.0083
ITPR3	1.3	0.21	1.6	0.042
ITGB1	1.6	0.029	1.8	0.013

$$\begin{array}{c} \text{Risk}\;\text{score}=(1.0167070\times \text{expression level of MET})\\ -(0.4313794\times \text{expression level of ENO3})\\ -(0.1764747\times \text{expression level of CD36}). \end{array}$$

With the median risk score as the cutoff, PDAC patients were classified into a low-risk group (n = 89) and a high-risk group (n = 88) (Figure 3C). Survival analysis confirmed that the survival times of the low risk patients were distinctly longer than those of the high-risk patients in the TCGA discovery cohort (P < 0.0001; Figure 3D). The finding was subsequently validated in ICGC validation datasets; the results obtained for these datasets agreed with those obtained for the discovery cohort (P = 0.038; Figure 3F). The same trend was also verified in the FUSCC validation cohort (P = 0.017; Figure 3H). Furthermore, to evaluate the performance of the three-gene signature in predicting the prognoses of PDAC patients, time-dependent ROC curves with respect to the 1-year, 3-year and 5-year survival rates were constructed for the TCGA, ICGC and FUSCC datasets, and the area under the curve (AUC) values for these curves were evaluated (Figure 3E, 3G, 3I). The AUC at 1, 3, and 5 years was 0.709, 0.749, and 0.836 in the TCGA set, respectively. The AUC at 1, 3, and 5 years was 0.644, 0.621, and 0.955 in the ICGC set, respectively. The AUC at 1 and 3 years was 0.601, 0.745 in the FUSCC set, respectively.

### Validation of the expression of and alterations in the three genes

**Figure 4:**
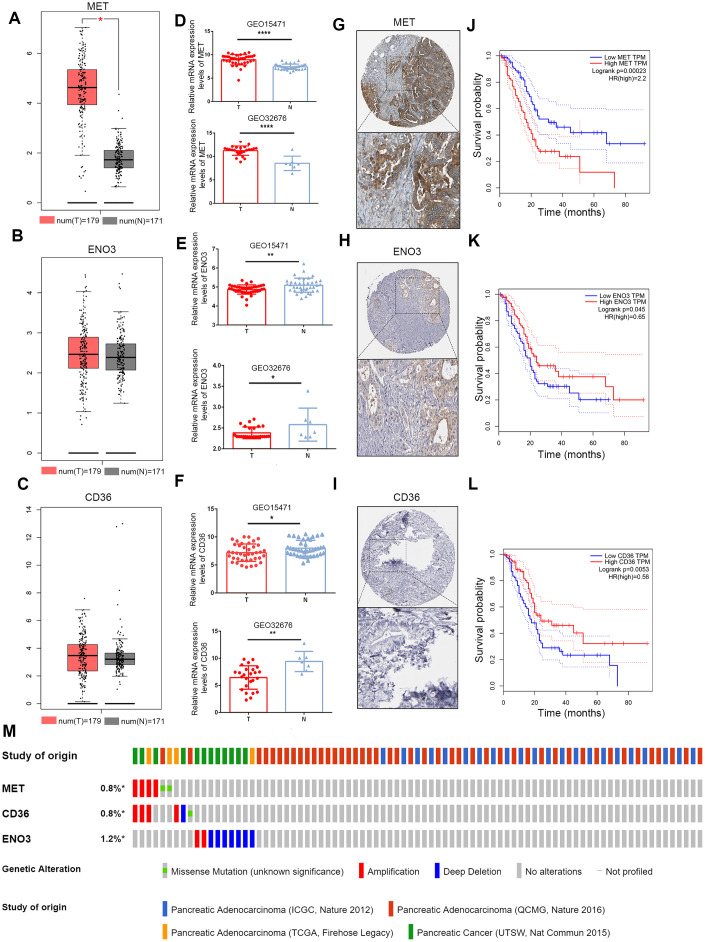
**Validation of expression and alteration of the three genes in pancreatic cancer.** (**A**–**C**) The MET, CD36 and ENO3 mRNA expression levels in TCGA pancreatic cancer tumor tissue and matching normal tissue from data on TCGA and GTEx. Data were obtained from the GEPIA (http://gepia.cancer-pku.cn/). (**D**–**F**) The MET, CD36 and ENO3 mRNA expression levels in GEO32676 and GEO15471 pancreatic cancer tumor tissue compared with non-tumor tissues. (**G**–**I**) The representative protein expression of the 3 glycolysis and lipid metabolism-based genes in pancreatic cancer tumor tissue. Data were obtained from the human protein atlas (https://www.proteinatlas.org/). (**J**–**L**) Survival analysis of patients with PAAD in terms of MET, CD36 and ENO3 in TCGA patients. (**M**) Genetic alterations of the three genes in the ICGC, QCMG, TCGA and UTSW pancreatic cancer datasets. Data were obtained from the cBioportal (https://www.cbioportal.org/).

The mRNA expression levels of the three genes were validated using the Gene Expression Profiling Interactive Analysis (GEPIA) database and GEO. In the GEPIA dataset, only MET mRNA expression was significantly elevated in tumor samples (Figure 4A–4C). In the GEO dataset, we found that MET expression levels were significantly higher in PDAC tumor tissue than in paracancerous tissue in the GEO32676 and GEO15471 datasets (Figure 4D). In contrast, the mRNA expression of ENO3 and CD36was markedly down-regulated in tumor tissues (Figure 4E, 4F). Protein expression of the DEGs in pancreatic tumor and non-tumor tissues was evaluated by the Human Protein Atlas (https://www.proteinatlas.org/) [12]. Immunohistochemical (IHC) staining information for the three genes in PDAC from the Human Protein Atlas database is shown in Figure 4G–4I. Moreover, the OS times were also obtained from the GEPIA database (Figure 4J–4L). A total of 848 patients in four cohorts (the ICGC, Queensland Centre for Medical Genomics [QCMG], TCGA and UT Southwestern Medical Center [UTSW] cohorts) were included in this study for analysis of the mutation information of the three glycolysis- and lipid metabolism-related genes. Overall, amplification and deep deletion were the most common types of mutations in MET, CD36 and ENO3 (Figure 4M).

### Validation of the risk score formula in the GEO and TCGA cohorts

**Figure 5:**
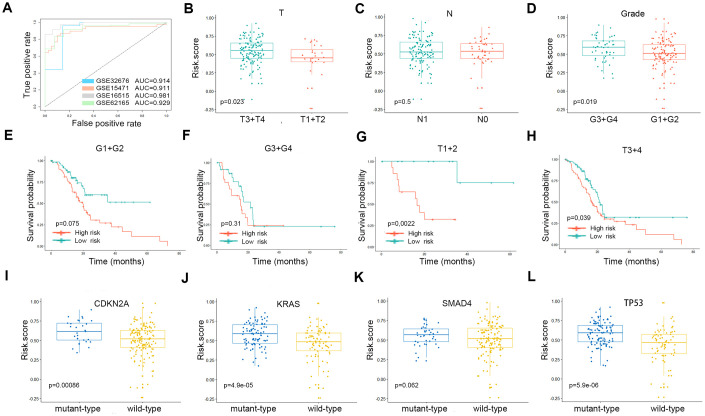
(**A**) The ROC curves of the risk scores differentiating pancreatic cancer from normal tissues in the four validation GEO datasets. The clinical and tumor mutation relevance of the three gene signatures. (**B**–**D**) The distribution of the risk scores in different AJCC stages in the TCGA cohort. (**E**–**H**) The Kaplan-Meier plot (low risk- score vs. high risk- score) of 5-year overall survival in patients in the TCGA cohort. (**I**–**L**) The expression level of the risk score in different mutation statuses of KRAS, TP53, CDKN2A, and SMAD4 in the TCGA dataset.

ROC curve analysis was performed with AUCs to evaluate the usefulness of the risk score of the gene signature in distinguishing PDAC tissues from control samples in four GEO cohorts (Figure 5A). Tumor tissues could be reliably identified based on this risk score. Subgroup analyses were performed to assess the three-gene signature and clinical characteristics of pancreatic cancer (including American Joint Committee on Cancer [AJCC] stage and grade) from appropriate datasets. In terms of AJCC stage and grade, stage T3-4 and grade 3-4 patients had higher risk scores than stage T1-2 and grade 1-2 patients. However, the same trend was not noted for N stages (Figure 5B–5D). The Kaplan-Meier curves of these clinical features are shown in Figure 5E–5H. The survival time of the high-risk score group was significantly shorter than that of the low-risk score group for all pathological T stages and for grades 1-2. In terms of mutations, risk scores were observed to be highly correlated with the mutation states of key genes in PDAC. The risk scores for the CDKN2A, KRAS and TP53 mutant groups were significantly higher than that of the wild-type group (Figure 5I–5L).

### Correlations between the three-gene signature and clinical characteristics

**Figure 6:**
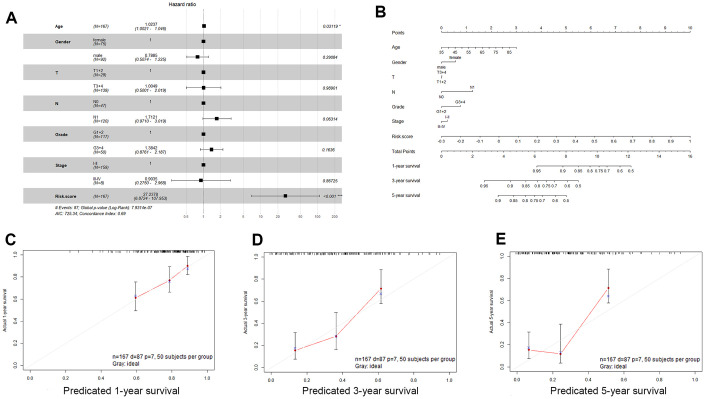
**Validation of the nomogram in predicting overall survival of pancreatic cancer in the TCGA dataset.** (**A**) Forest plot summary of multivariable Cox regression analyses of the risk score, age, sex, grade and tumor stage in the TCGA cohort. The squares represent the hazard ratio (HR), and the transverse lines represent 95% CIs. CI, confidence interval. (**B**) A nomogram to predict survival probability at 1, 3 and 5 years for PDAC patients based on the results derived from the TCGA cohort. (**C**–**E**) Calibration curve for the nomogram when predicting 1, 3- and 5-year overall survival.

We examined the association of the three-gene signature (risk score) with clinical features in PDAC patients using univariate and multivariate Cox proportional hazard regression analyses. Univariate Cox proportional hazard regression showed that N stage, age, and risk score could predict poor survival of PDAC patients, as shown in Table 2 (P < 0.05). In addition, multivariate Cox proportional hazard regression showed that the risk score (P < 0.001) was an independent prognostic indicator of PDAC (Figure 6A). Based on the results acquired from multivariate Cox regression of OS in the TCGA dataset, we developed a nomogram to predict 1-, 3- and 5-year survival probability in PDAC (Figure 6B). The C-index of our nomogram in the TCGA cohort was 0.689 (95% confidence interval, 0.628-0.749), and the calibration curves for this nomogram presented good agreement between the possibility estimated by the nomogram and the actual proportion (Figure 6C–6E).

**Table 2:** Univariable Cox regression analyses of the risk score, age, gender, grade and tumor stage TCGA.

	**HR (95% CI for HR)**	**Wald. Test**	**P. value**
Age	1 (1-1.1)	5.4	0.02
Gender	0.8 (0.53-1.2)	1	0.31
Risk. Score	31 (8-120)	24	8.60E^-07^
Grade	1.5 (0.93-2.3)	2.8	0.097
Stage	0.74 (0.23-2.4)	0.26	0.61
N	2.1 (1.3-3.6)	7.9	0.0049
T	1.8 (0.93-3.5)	3	0.081

### Analysis of gene set enrichment relevance of the three-gene signature

**Figure 7:**
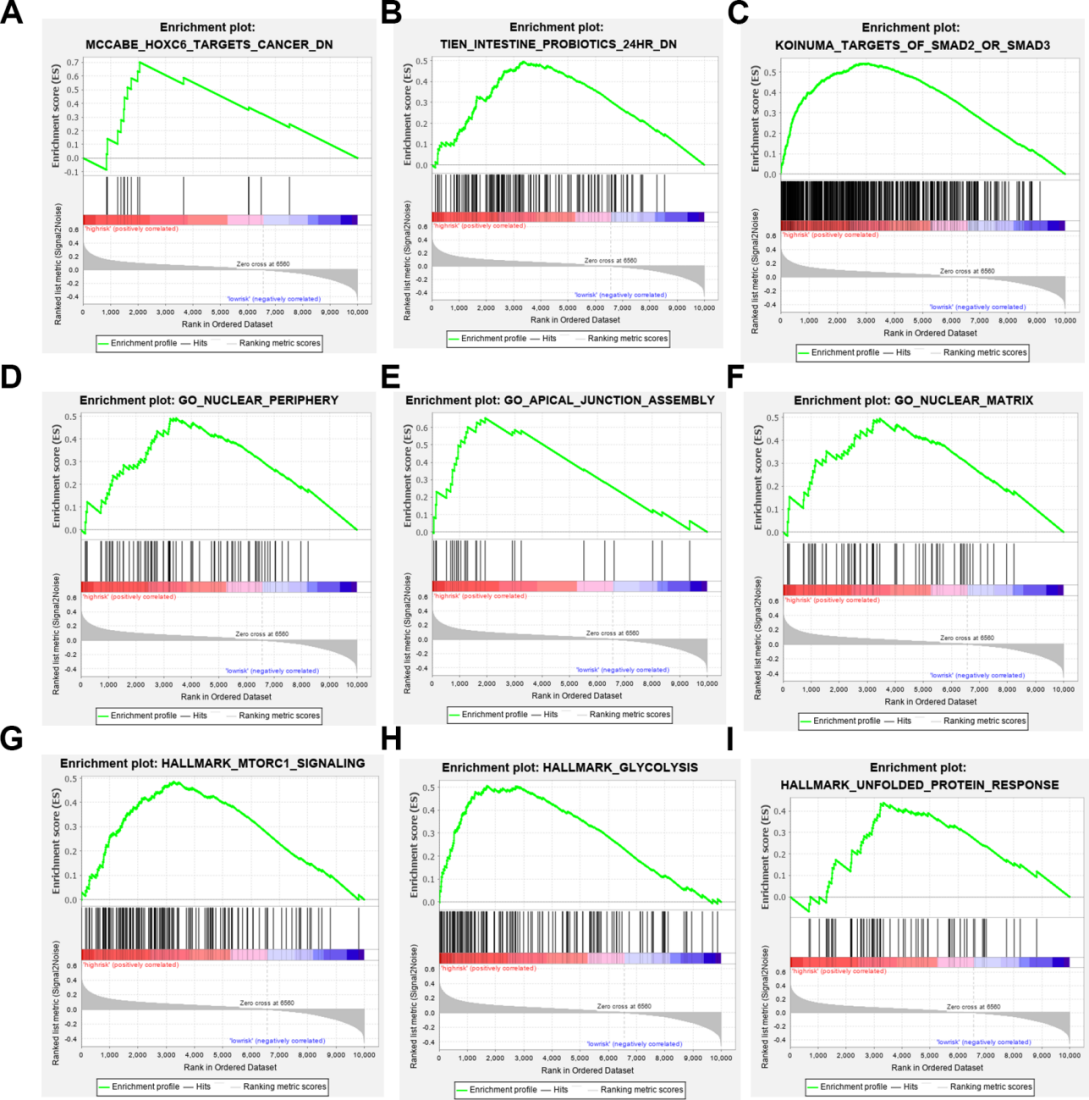
**Gene set enrichment analyses.** (**A**–**C**) Top 3 (HOXC6 target cancer, intestine probiotics, target of SMAD2 or SMAD3.) oncological signatures significantly C2 (hallmark gene sets) enriched in the high-risk group identified by gene set enrichment analysis. (**D**–**F**) Top 3 (nuclear periphery, apical junction assembly, nuclear matrix.) oncological signatures significantly C5 (biological process) enriched in the high-risk group identified by gene set enrichment analysis. (**G**–**I**) Top 3 (MTORC1 signaling, glycolysis, unfolded protein response.) oncological signatures significantly H (hallmark gene sets) enriched in the high-risk group identified by gene set enrichment analysis.

To elucidate the molecular mechanism of the three-gene signature, patients from the TCGA PDAC dataset were divided into high- and low-risk groups according to the cutoff value. GSEA compared the high- and low-risk groups. Gene ontology (GO) gathers information about molecular function, biological processes, and cellular components in a number of different organisms. These enriched gene ontology (GO) data revealed that molecular alteration in the high-risk group was closely related to the nuclear periphery, apical junction and nuclear matrix. Oncological signatures were significantly enriched, including HOXC6, SMAD2, SMAD3 and MTORC (Figure 7).

## DISCUSSION

Pancreatic ductal adenocarcinoma (PDAC) is one of the most invasive solid malignancies and could become the second leading source of cancer-related deaths in the United States by 2030 [13]. Therefore, updated models with effective gene signatures are needed that can better identify patients with PDAC in high- and low-risk groups. Such models could aid in the selection of appropriate treatments and improve clinical management. Currently, clinical indices and tumor biomarkers are used to predict prognosis and relapse in patients with PDAC [14]. However, the value of some clinical features, such as tumor dimensions and lymph node metastases, in relapse and survival prediction remains controversial. Carbohydrate antigen 19-9 (CA19-9), CA50 and carcinoembryonic antigen (CEA) are classic serum biomarkers used for PDAC prognosis prediction. However, these tumor markers are not specific to PDAC; thus, elevations in their levels can be related to other gastrointestinal carcinomas. Compared with most solid cancers, PDAC is recognized as a more heterogeneous disease influenced by genetic polymorphisms and hypoxic environments, and metabolic reprogramming is a novel feature of PDAC. It has become increasingly recognized that extensive enhancement of glycolysis and reprogramming of lipid metabolism are both associated with the development and progression of PDAC [15]. Previous studies have suggested that various gene signatures could convey prognostic information about PDAC. However, the use of these signatures has some limitations, perhaps because of a lack of knowledge regarding the genetic and energy supply backgrounds of PDAC. Therefore, we conducted multi-mRNA analysis based on metabolic reprogramming to identify novel signatures for accurate prognosis prediction in PDAC patients.

In the current study, a three-gene signature based on metabolic reprogramming was identified using the LASSO Cox regression model. This signature offered excellent accuracy in identifying patients with poor survival. The signature also showed significant ability to distinguish between PDAC and non-PDAC in GEO datasets and a strong, positive correlation with the mutation states of key genes in PDAC. Univariable and multivariate Cox analyses indicated that the three-gene signature was a powerful and independent prognostic indicator. Additionally, we developed a nomogram that included clinical factors. GSEA was used to further explore the underlying mechanism of the signature in high-risk PDAC patients.

PDAC cells have a powerful ability to survive in harsh environments by transforming their energy metabolism processes. MET, also known as c-MET, is a receptor tyrosine kinase that builds connections between the extracellular matrix and cytoplasm by binding with its ligand hepatocyte growth factor (HGF). The MET receptor incorporates its ligand, HGF, through MET dimerization, and this process activates MET. In cancer cells, aberrant HGF/c-Met axis triggering, which is strongly linked to c-Met gene mutations and amplification, promotes tumor development/progression by inducing the PI3K/AKT, Ras/MAPK, JAK/STAT, SRC, Wnt/β-catenin, and other signaling pathways. Up-regulated MET expression is also related to a shorter time to distant metastasis in PDAC patients receiving adjuvant and neoadjuvant chemoradiation therapy [16]. Furthermore, Yan et al. revealed a novel link between paracrine HGF/c-MET signaling and enhancement of stem cell-like potential and glycolysis in PDAC, involving activation of yes-associated protein [17]. Currently, drugs targeting c-Met that are in clinical trials can be divided into monoclonal antibodies (e.g., onartuzumab) and small molecule inhibitors, including ATP-competitive inhibitors (e.g., crizotinib) and non-ATP competitive inhibitors (e.g., tivantinib). Targeting of c-Met for the treatment of tumorigenesis and metastasis is still believed to have broad clinical significance [18]. Enolase (ENO), also known as phosphopyruvate hydratase, is a metalloenzyme that catalyzes the conversion of 2-phosphoglycerate (2-PG) into phosphoenolpyruvate (PEP) during glycolysis and reverses the transformation of phosphoric acid-pyruvate into 2-phosphate-d-glycerate through glycogen synthesis. The ENOs in mammalian cells are composed of 3 subunits: α, or nonneuronal ENO (NNE); β, or muscle-specific ENO (MSE); and γ, or neuron-specific ENO (NSE) [19]. These molecules are cytoplasmic enzymes associated with glycolysis and gluconeogenesis. ENO1 is activated by various glucose transporters and glycolytic enzymes associated with cell cycle progression and the Warburg effect in tumor cells. Some studies have shown that ENO1 acts as a potent promoter in tumor cells by regulating AMPK/mTOR and PI3K/AKT signaling and inactivating a downstream signaling pathway [20]. Up-regulated expression of the ENO2 gene has also been observed in non-small cell lung carcinomas (NSCLCs). The β-subunit of ENO is encoded by the ENO3 gene. Choa Park et al. suggested that up-regulated ENO3 gene expression is directly positively correlated with the consequence of STK11 loss of function in lung cancer. Additionally, down-regulation of ENO3 expression has been found to exert a selective anticancer effect in STK11 mutant cells compared with STK11 wild-type cells. However, the role of ENO3 in PDAC remains unknown [21].

Fatty acid (FA) derivatives consist of sterols, phospholipids, and sphingolipids, along with signaling molecules that mediate the invasion, migration, and chemoresistance of cancer cells [7]. CD36, a transmembrane glycoprotein that belongs to the scavenger receptor class B family, is an essential member of this group [22]. In addition to FA uptake, CD36 promotes cholesterol uptake and transduces intracellular signaling, mediating the metabolic targeting of FAs. CD36 has also been revealed to play roles in other important cellular processes related to tumor biology, including angiogenesis, antigen presentation and cell adhesion [23]. Recent reports have revealed that low expression of CD36 is associated with low TNM stages and CA19-9 levels but poor survival prognosis in PDAC patients [24]. However, Masahiko Kubo et al. suggested that increased expression of CD36 could increase gemcitabine resistance by regulating antiapoptotic proteins and the endothelial-mesenchymal transition of PDAC cells [25]. These controversial results rendered the role of CD36 in PDAC unclear and could be partly due to limited awareness of FA components and their contributions to the biological behavior of PDAC.

Like other studies, the present study inevitably had some limitations. First, the main sources of our research data were public datasets, and our findings were not validated in a prospective cohort. Moreover, more experiments incorporating multiplatform analyses with genomics, transcriptomics and proteomics approaches should be performed to investigate the molecular mechanism of the identified signature to illuminate the association between the three-gene signature and PDAC prognosis.

In conclusion, our study successfully identified a three-gene signature and a prognostic nomogram for the prediction of OS in pancreatic cancer patients. The three-gene signature was found to be closely associated with the progression and prognosis of pancreatic cancer. This classifier could have clinical applications in guiding the use of metabolic reprogramming and could be useful for the personalized management of PDAC patients. Future investigations of the related molecular mechanisms and prospective, randomized clinical trials regarding this signature will be of great clinical significance and could provide a roadmap for precision medicine.

## MATERIALS AND METHODS

### Acquisition of PDAC gene expression data and clinical data

Microarray gene expression profiles for PDAC were downloaded from the NCBI Gene Expression Omnibus (GEO) (https://www.ncbi.nlm.nih.gov/geo/), TCGA (http://cancergenome.nih.gov/) and the International Cancer Genome Consortium (ICGC) database (https://dcc.icgc.org/). The gene expression microarray datasets GSE15471, GSE16515 and GSE32676 were chosen and downloaded for differentially expressed gene (DEG) analysis. The datasets met the following criterion: they contained data on human pancreatic tissue samples from tumors and adjacent paracancerous areas. Datasets that were unanalyzable, that included few DEGs (fewer than 100), or that included defectively annotated genes were excluded. The probe names were transformed into gene symbols using the annotation files supplied by the manufacturer. The median ranking value was used as the expression value if several probes matched a single gene. Robust multiarray average (RMA)-normalized data were log2-transformed for further analysis. Normalized RNA-sequencing data in transcripts per million (TPM) format and the associated clinical information of the PDAC samples were downloaded from the TCGA dataset. For validation, we used transcriptomic data from the ICGC and recruited 82 patients who underwent surgical resection at Fudan University Shanghai Cancer Center (FUSCC) from 2010 to 2012. These patients were diagnosed according to strict pathological criteria, and total RNA was extracted from their tumor tissues. Strictly controlled postoperative follow-ups were conducted for all of the patients. All of the procedures were implemented after obtaining approval from the Clinical Research Ethics Committee of FUSCC, and informed consent was acquired from each patient.

### DEG identification and bioinformatic analysis

The “limma” package of R software (version 3.6.2) was used to establish a prognostic gene signature by identifying the DEGs from the mRNA data. A |log2(fold change [FC]) | > 1, a P-value < 0.05, and a false discovery rate (FDR) < 0.05 were considered the cutoffs for DEGs between tumor and nontumor samples in the GEO database. Next, the DEGs that overlapped among 3 series, as determined with Venn and UpSet diagrams, were regarded as credible DEGs. Gene ontology (GO) enrichment and Kyoto Encyclopedia of Genes and Genomes (KEGG) pathway analyses were applied to explore the potential biological roles of the DEGs. Significantly relevant signaling pathways were identified with the Database for Annotation, Visualization, and Integrated Discovery (DAVID) (https://david.ncifcrf.gov/) [26]. P < 0.05 was considered the threshold for statistical significance.

### Establishment of the LASSO regression model and calculation of risk score

We used log-rank tests to examine the associations between gene expression and OS in the TCGA dataset. DEGs with P-values < 0.05 were considered statistically significant and were included in the following analyses. Genes obtained from the Molecular Signature Database (MSigDB) gene sets “REACTOME_GLYCOLYSIS” and “REACTOME_METABOLISM_OF_LIPIDS_AND_LIPOPROTEINS” were used as glycolytic and lipid metabolism-related genes, respectively [7, 8, 27]. Twelve glycolysis and lipid metabolism-related genes were selected to construct LASSO Cox regression analysis models with the R package “glmnet”. LASSO analysis is the most popular method for evaluating survival data and is especially appropriate for analyzing gene expression profiles. The “glmnet” package returned a series of models. For each model, the tuning parameter λ was inversely correlated with the complexity of the model and the value of deviance. Ten-fold cross-validation was performed to screen the best model from the series (the model with the minimum mean cross-validation error). The patients were divided into high-risk and low-risk groups according to a cutoff median risk score. Kaplan-Meier analysis and time-dependent receiver-operating characteristic (ROC) curve analysis were used to test predictive differences between the high- and low-risk groups in the discovery and two validation datasets. Cox regression analysis was employed to test whether the risk score was an independent prognostic factor. A nomogram and related calibration curves were created based on the TCGA cohort for further clinical application.

### Gene set enrichment analysis (GSEA) and mutation analyses

GSEA was applied to elucidate the molecular mechanisms of the prognostic gene sets [28]. The TCGA dataset was divided into high- and low-risk groups according to the cutoffs. GSEA was performed in the Java program GSEA, version 3.0. The MSigDB v. 6.2. C2 collection (curated gene sets), C5 collection (GO gene sets), and H collection (hallmark gene sets) were searched to identify enriched KEGG pathways and biological information and dysfunctional oncogenic signatures associated with the high-risk group. Results with a |normalized enrichment score (NES)| > 1 and an FDR < 0.05 were considered statistically significant. Somatic mutations (SNPs and small indels) in TCGA were obtained with MuTect2 in the University of California, Santa Cruz (UCSC), with the Xena browser (https://xenabrowser.net/datapages/). Genetic alterations in the three genes associated related to pancreatic cancer were obtained from cBioPortal (https://www.cbioportal.org/) [29].

### RNA extraction, reverse transcription, and qRT-PCR analysis

RNA was obtained from patients’ samples and clinically diagnosed with PDAC at the Fudan University Shanghai Cancer Center (FUSCC) from 2010 to 2012. Informed consent was obtained from each patient, and all of the experiments were performed with the approval of the Clinical Research Ethics Committee of FUSCC (ethical code: 050432-4-1212B). In the FUSCC validation set, total RNA was extracted from 82 patient samples using a MiniBEST Universal RNA Extraction Kit (9767, TaKaRa). A PrimeScript RT Reagent Kit (K1622, Thermo Scientific) was used to synthesize first-strand cDNA isolated from total RNA. Then, SYBR Green qRT-PCR was conducted on an ABI 7900HT platform (Applied Biosystems, USA). We used GAPDH mRNA as an internal reference. The primers used for the mRNA molecules tested in this study were synthesized by Sangon (Shanghai, China), and the sequences are listed in Table 3.

**Table 3:** The sequences of the primers for the three genes signatures.

**Primer**	**Sequence (5'to 3')**	**Base number**
m-MET-F	AGCAATGGGGAGTGTAAAGAGG	22
m-MET-R	CCCAGTCTTGTACTCAGCAAC	21
m-ENO3-F	GGCTGGTTACCCAGACAAGG	20
m-ENO3-R	TCGTACTTCCCATTGCGATAGAA	23
m-CD36-F	GGCTGTGACCGGAACTGTG	19
m-CD36-R	AGGTCTCCAACTGGCATTAGAA	22

### Statistical analysis

Statistical analysis was performed in R software (version 3.6.2, https://www.r-project.org/) and GraphPad Prism software, version 7.01 (GraphPad Software, La Jolla, CA, USA). Categorical variables were analyzed by the χ^2^ test or Fisher’s exact test. Continuous variables were analyzed using Student’s t-test for paired samples. Kaplan-Meier curves and log-rank tests were used. Calibration plots were produced to assess whether actual outcomes matched predicted outcomes for the nomogram. All of the statistical tests were 2 sided, and a P-value < 0.05 was considered to indicate statistical significance.
